# Expression of miRNAs (146a and 155) in human peri-implant tissue affected by peri-implantitis: a case control study

**DOI:** 10.1186/s12903-024-04579-x

**Published:** 2024-07-28

**Authors:** Munir Nasr Hamed, Hayder Raad Abdulbaqi

**Affiliations:** 1 Department of Periodontics, College of Dentistry, University of Baghdad, Baghdad, Iraq; 2 Department of Dentistry, Dijlah University College, Baghdad, Iraq

**Keywords:** Humans, Peri-implantitis, microRNAs, Dental implant, qPCR, RNA

## Abstract

**Background:**

In literature, the levels of miRNA-146a and miRNA-155 are increased in periodontitis. Limited data are available regarding the expression of miRNA-146a and miR-NA-155 in diseased human peri-implant tissue. Therefore, the objective of this study was to explore the expression of miRNA-146a and miRNA-155 in human gingival peri-implant tissue affected by peri-implantitis.

**Methods:**

After recording the clinical parameters, human peri-implant pocket tissues were harvested from sites diagnosed with peri-implantitis (*n* = 15 cases) in addition to healthy peri-implant sulcus tissues (*n* = 15 controls). The levels of miRNA-146a and miRNA-155 were assessed using real-time qPCR.

**Results:**

Cases exhibited a significantly higher mean expression of miRNA-155 (5.2-fold increase) and miRNA-146a (2.8-fold increase) than controls. MiRNA-155 and miRNA-146a demonstrated an appropriate sensitivity (87.5% and 87.5%, respectively) and specificity (73.3% and 66.7%, respectively) in discriminating cases from controls. A moderate correlation (*r* = 0.544, *p* = 0.029) was found between miRNA-155 and miRNA-146a levels in the case group.

**Conclusions:**

The expressions of miRNA-146a and miR-NA-155 are different between healthy and peri-implantitis affected tissues. Both miRNAs might potentially able to discriminate healthy from peri-implantitis affected tissues.

**Supplementary Information:**

The online version contains supplementary material available at 10.1186/s12903-024-04579-x.

## Background

Peri-implantitis is a biofilm-induced inflammation of the oral mucosa surrounding dental implants. Clinically, it is characterized by increased peri-implant pocketing, bleeding gingiva, and loss of alveolar bone [1, 2]. Peri-implantitis is a widespread disease worldwide; its prevalence is 19.53% at the patient level and 12.53% at the implant level [3]. Treatment of peri-implantitis mostly needs surgical intervention [4]. Unfortunately, the success of peri-implantitis therapy is uncertain, particularly in cases with severe bone loss [5–7]. Therefore, early detection of pathological peri-implant bone resorption is of great importance. Nowadays, the role of biomarkers for early identification and accurate prediction of peri-implant diseases is getting much attention [8–10].

In peri-implant diseases, local inflammatory responses are generated and maintained in the peri-implant tissue by the interactions of cytokines and chemokines secreted from activated lymphocytes, macrophages, and neutrophils [11–13]. Peri-implant bone loss is the consequence of prolonged local inflammation that leads to pathological bone remodeling and, consequently to implant failure [14]. Earlier studies have shown that miRNAs (miRNAs) play a critical role in numerous inflammatory diseases [15, 16] such as periodontitis [17–19]. Abnormal expression of these molecules leads to various systemic and oral disease [20–23]. MiRNAs are small (18–22 base pairs) non-coding RNA molecules [24, 25]. They participate in regulating gene expression [26]. MiRNAs induce degradation or suppression of the targeted messenger RNA molecule after binding to its complementary sequence of the 3-UTR (untranslated coding region) [27–30]. Among miRNAs, miRNA-146a and miRNA-155 have been reported to be stimulated by Toll-like receptors (TLRs) and proinflammatory cytokines and have a regulatory role in immune reactions [31, 32].

The expression of both miRNA-146a and miRNA-155 have been extensively investigated in periodontitis patients. As compared to subjects with healthy periodontium, periodontitis patients have been reported to show higher expression of miRNA-146a [18, 33, 34]. This finding is further supported by a recent meta-analysis which also has suggested that miRNA-146a might have a potential role in discriminating periodontal health from disease [35]. At the same time, miRNA-155 shows a similar trend as it has been demonstrated to be upregulated in periodontitis compared to periodontal health [29, 33]. However, a contrary finding (downregulation of miRNA-155) has also been reported in periodontitis [34]. This suggests that many miRNAs might demonstrate a dual regulation effect. Such behavior could be explained by the ability of a single miRNA to impact and control many genes [36]. Therefore, more research is needed to determine the significance of this dual regulation effects of miRNAs in the regression and progression of diseases. On the other hand, no data are available about the expression of both above-mentioned miRNAs in peri-implantitis. Only one animal study that have revealed a considerable increase in expression of miRNA-146 [14]. Despite similarities between periodontitis and peri-implantitis conditions with respect to etiology and pathogenesis, more pronounced inflammatory responses are encountered to bacterial challenges in peri-implant tissue [37, 38]. There are distinct molecular and histopathological characteristics of peri-implantitis compared to periodontitis, despite some similarities between the two conditions [39]. Therefore, this study aimed to explore the expression level of miRNA-146a and miRNA-155 in human gingival tissue surrounding dental implants diagnosed with peri-implantitis and health.

## Materials and methods

### Study design

This case-control study was carried out between July 2022 and August 2023. The peri-implant tissue samples were collected from patients attending several private clinics in Baghdad. The study protocol was earlier approved by the ethical committee of the College of Dentistry / University of Baghdad in April 2022 (project No.#534,622). All selected patients signed informed consents which were collected by the study examiner.

### Sample size

In this study, the sample size was calculated after conducting a pilot study involving 6 tissue samples; three diseased and three healthy tissue samples. After processing the tissue samples, the estimated mean fold changes of miRNA-155 (primary outcome) were 0.15 ± 0.11 and 0.45 ± 0.44 in healthy and diseased tissues, respectively; the estimated effect size was 0.935. Accordingly, it was found that, at least, a total size of 30 tissue samples (15 for each group) was enough to reject the null hypothesis at a power of 80% and 0.05 probability. G*power 3.1 software was used to calculate the sample size.

### Inclusion and exclusion criteria

Tissue samples were collected from patients who were seeking to replace missing teeth with dental implants (control group) and patients already having dental implants diagnosed with peri-implantitis (case group). To include patients in this study, they should be systemically healthy adult (> 18 years). The selection of patients in the control group was based on the adequacy of their bone quality and quantity for implant placement. A minimum of 8.5–13 mm bone length and 4.5–5 mm bone width were the thresholds for selecting patients in this group. In addition, the selected patients should have a minimum 3 mm width of keratinized mucosa. While for case group, patients should have at least one implant with more than one year in function diagnosed with peri-implantitis. The considered implants should have bleeding and/or suppuration on gentle probing, increased probing pocket depth (PPD) compared to previous baseline data and bone loss beyond initial bone remodeling levels. In the absence of previous examination data, the included diagnosed implants should exhibit bleeding and/or suppuration on gentle probing, PPD of ≥ 6 mm and bone levels ≥ 3 mm apical to the most coronal portion of the intraosseous part of the implant [1]. The exclusion criteria included positive history of periodontitis, active infectious diseases, cardio-vascular diseases, type I or type II diabetes and receiving local or systemic anti-inflammatory/antimicrobial treatment 3 months before starting the study.

### Clinical examination

The eligible patients were clinically examined to detect any signs of mucosal inflammation, such as redness or edema, and measure PPD and bleeding on probing (BOP) sites followed by digital radiographic imaging to verify the presence of osseous resorption. All clinical parameters were measured by a University of North Carolina-15 (UNC) periodontal probe.

For each site, the periodontal probe was inserted gently into the pockets until feeling resistance then removed. After 15 s, the presence or absence of BOP in the sites were recorded as 1 or 0 respectively [40]. The presence of suppuration in the peri-implant sites was detected through palpation by fingers of oral and facial peri-implant tissues. The presence or absence of suppuration was recorded as 1 or 0 respectively [40]. PPD was measured by periodontal probe as the distance between the margin and bottom of the peri-implant pocket [41].

The bone loss was determined by measuring the length from the bone margin to the platform of the implant on periapical digital x-ray using the true length of the implant fixture as a reference. The x-rays were processed with dental x-ray software to make linear measurements. The actual size of the bone defect at the implant site was obtained by adjusting the implant relative size as following: Linear distance in mm equals (true implant size * mesial or distal length at x-ray / implant size in x-ray). The mesial or distal surface true values were obtained [42]. The width of keratinized gingiva was assessed as previously reported [43]. While, the gingival thickness was assessed using an endodontic reamer (#15) with a rubber stopper. First, the site was anesthetized, and then the reamer was inserted 2 mm apical to the gingival margin at mid-buccal sites. The stopper was adjusted to mark the depth of insertion, and the length of the reamer from the tip to the stopper was measured by a digital caliper [44].

### Tissue sampling

All patients in control group had received dental implants (Medentika, Germany). The surgical implant placement was done by one experienced surgeon (M.P) for all patients. Amoxicillin (2 g/day, GlaxoSmithKline, UK) was prescribed for patients for 6 days. Sutures were removed after a period of 7–10 days [45].

After 12 ± 4 weeks healing period, the dental implants were surgically exposed by the same surgeon to replace cover screws by narrow healing abutments. All patients received oral hygiene instructions for full mouth-mouth cleaning including brushing and interdental cleaning twice daily. Subsequently, after one month of healing period, patients came again to clinic in order to collect healthy tissue samples. The peri-implant tissue was examined to confirm the lack of bleeding with gentle probing and the absence of redness to avoid any confusion with mucositis. The clinical parameters were recorded before peri-implant tissue harvesting. In this visit, a circular incision, after giving local anesthesia, was made by the same surgeon using a scalpel blade (15 C, Aesculap AG) or tissue punch. By using tissue forceps and scalpel 1.5 mm of peri-implant gingival tissue was obtained around each abutment. Then, a larger diameter healing abutment was screwed into the implant as shown in Figure S1 [45–47]. Patients were re-instructed about oral hygiene measures at this visit.

For patients in the case group, diseased peri-implant tissue samples were harvested as a part of their surgical treatment after recording the clinical parameters. After anesthetizing the area, two parallel incisions, 3 mm apart, were made through the soft tissue (peri-implant pocket sites) using a micro-scalpel blade (MEDESY CO, Italy) until reaching bone at proximal side of the dental implant. The two incisions were joined by a perpendicular incision 4 mm from the implant proximal area. Then specimens, which included the entire supra-crystal soft tissue part of the affected location, were carefully detached using tissue forceps and scalpel (MEDESY CO, Italy) [46, 47].

All tissue samples were rinsed with normal saline, preserved in a 2 ml tube containing DNA/RNA Shield (Zymo Research Europe, Freiberg, Germany) to avoid tissue degradation (preserving DNA/RNA intact). These tubes were immediately saved in a transfer cooling box (at 4 °C) for one hour and then saved at -20 °C in a refrigerator until RNA extraction (after 6 months).

### Total RNA extraction and quality control

The saved frozen tubes containing tissue samples were left for 5 min at room temperature, vortexed, and then centrifuged at maximum speed for two minutes. The tissue was accumulated as a pellet at the bottom of the tube after aspirating the shield solution (supernatant layer). For homogenizing the tissue and isolation of total RNA, QIAzol™ Reagent kit was used according to manufactural instructions with the aid of 1.0 mm fused silica beads (BioSpec, USA) as shown in Figure S2.

The total RNA quality and quantity of the homogenized tissue samples was assessed using a spectrophotometer (Nabi-UV/VIS Nano Spectrophotometer Micro-Digital Co., Ltd., Seoul, Korea) [48].

### RNA reverse transcription and quantification of miRNAs

The total RNA was converted to complementary DNA (cDNA) using miRNA-specific stem-loop primers (GoScript Reverse Transcription System, Promega, USA) according to the manufacturer instructions. The used primers are listed in Table 1. Quantus-Fluorometer (Promega, USA) was used to estimate the concentration of extracted cDNA. The levels of miRNA-155 and miRNA-146a gene were quantified using GoTaq^®^ qPCR Master Mix (Promega, USA) kits based on the manufacturer instructions by real-time polymerase chain reaction (qPCR) device (Mic-PCR-Cycler, Bio-Molecular, Australia). Briefly, the Real-Time qPCR was started with an initial denaturation step at 95 °C for 5 min, followed by 40 cycles of denaturation at 95 °C for 15 s, annealing at 55 °C for 15 s, and extension at 72 °C for 15 s. The program ends with a final hold step at 4 °C for 10 min. The fluorescence signals were acquired during the annealing step to detect the amplification of the gene.

**Table 1:** Primers used in this study

Primer *	Sequence 5’-3’	Annealing Temp. (ºC)
miRNA-146a-5p-RT	GTTGGCTCTGGTGCAGGGTCCGAGGTATTCG CACCAGAGCCAACAACCCA	55 ºC
miRNA-146a-5p-F1	GTTTGGTGAGAACTGAATTCCA	
miRNA-155-5p-RT	GTTGGCTCTGGTGCAGGGTCCGAGGTATTCG CACCAGAGCCAACACCCCT	
miRNA-155-5p-F1	GTGGGTTAATGCTAATCGTGAT	
RNU43-RT	GTTGGCTCTGGTGCAGGGTCCGAGGTATTCG CACCAGAGCCAACAATCAG	
RNU43-F	GTGAACTTATTGACGGGCG	
Universal Reverse prime	GTGCAGGGTCCGAGGT	

^**1**^ These specific primers were designed at (Primer3 software, https://primer3.org/releases.html), supplied by Macro-gen Company in a lyophilized form

The 2−∆∆CT method was used to estimate the relative expression of miRNAs [49] as following:

ΔCT = CT gene - CT Housekeeping gene.

ΔΔCT = ΔCT Treated or Control – Average ΔCT Control.

Fold change = 2-ΔΔCT.

### Statistical analysis

The data showed in Table S1 were analyzed using SPSS software (V.26; Chicago, IL, USA). The SPSS output draft is shown in Table S2. The normal distribution of numeric variables was detected by exploring their skewness. Each variable showed skewness < -1 or > 1 was considered as non-normally distributed. Mann Whitney U test was used to compare between non-normally distributed variables. On the other hand, Student t-test was used to detect any difference between normally distributed variables. The receiver operator curve [46] was used for checking the reliability criteria and the cutoff values of miRNAs (miR155 and miR146a) in detecting diseased tissue. The considered level of alpha for significance was < 0.05.

## Results

**Fig. 1:**
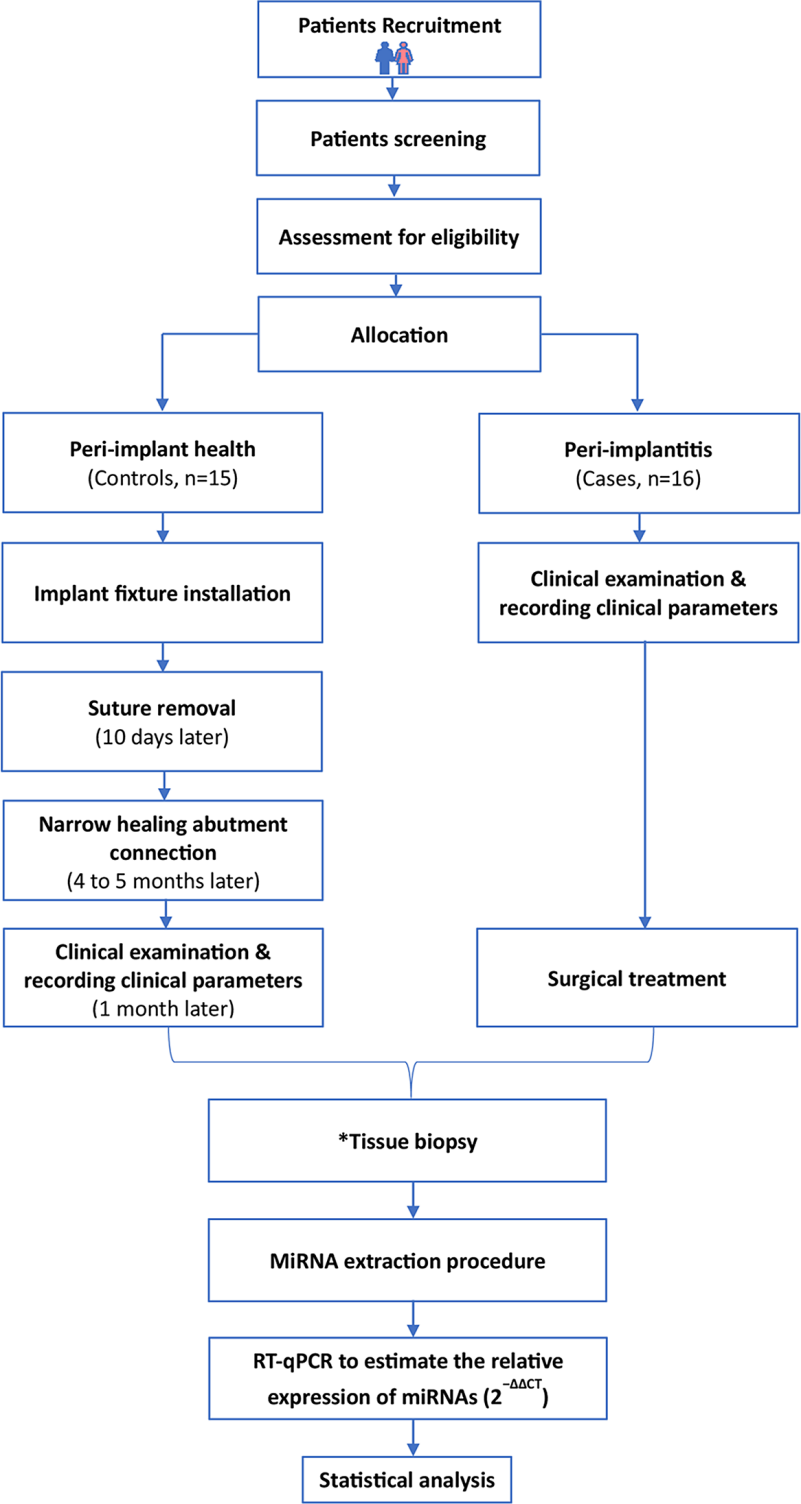
Flow chart of the study

Peri-implant tissue samples were harvested from 16 (44.8 ± 11.1 years) and 15 (49.1 ± 9.7 years) volunteers in the case and control groups respectively as shown in Fig. 1. The sex distribution was not statistically different between the groups (*p* = 0.095). Means of PPD and gingival width were significantly higher in case than control groups (*p* < 0.001 and *p* = 0.004, respectively). While no difference was detected in mean gingival thickness between the groups (see Table 2). Means of BOP and bone loss as well as suppuration frequency in the case group are shown in Table 2.

**Table 2:** Basic variables of the study groups

	Case *n* (%)	Control *n* (%)	*p*-value
**Sex**			
Male	9 (56.3%)	4 (26.7%)	> 0.05^c^
Female	7 (43.8%)	11 (73.3%)	
Suppuration per surface	24 (25%)	0	NA
	**Mean ± SD**	**Mean ± SD**	
Age (years)	44.8 ± 12.1	49.1 ± 9.7	> 0.05^b^
BOP	0.8 ± 0.2	0	NA
PDD (mm)	5.6 ± 0.7	1.9 ± 0.4	**< 0.05** ^a^
Gingival Width (mm)	5.6 ± 2	4 ± 0.4	**< 0.05** ^a^
Gingival Thickness (mm)	1.9 ± 0.4	1.9 ± 0.4	> 0.05^b^
Bone loss (mm)	3.6 ± 0.8	0	NA

^a^ Comparison by Student t-test. ^b^ Comparison by Mann-Whitney U test. ^c^ Comparison by X2 test. Significance level at p value < 0.05 (bold)

In this study, a significantly (*p* < 0.05) higher fold change of miRNA-155 (12.5 ± 14.2) was detected in tissue of cases than control group (2.4 ± 3.4). It was estimated that the average fold change of miRNA-155 in case group was 5.2 times higher than controls. Similarly, the fold change of miRNA-146a was found to be significantly (*p* < 0.05) higher in case group (8.6 ± 11.5) than control group (3.0 ± 4.3). Approximately, the fold change of miRNA-146a in case group was 2.8 times higher than control group as shown in Table 3.

**Table 3:** Mean level and fold change of miRNAs in the study groups

	Case Mean ± SD			Control Mean ± SD			Average fold change	*p* value*
	DCT (∆CT)	DDCT (∆∆CT)	Fold change (2^−∆∆CT^)	DCT (∆CT)	DDCT (∆∆CT)	Fold change (2^−∆∆CT^)	Mean case/mean control	
miRNA-155	-1.1 ± 2	-2.6 ± 2	12.5 ± 14.2	1.5 ± 2.1	0 ± 2.1	2.4 ± 3.4	5.2	< 0.05
miRNA-146a	-3.0 ± 2	-3.01 ± 2	8.6 ± 11.5	-1 ± 2.4	-1 ± 2.4	3.0 ± 4.3	2.8	< 0.05

* Comparison by Mann-Whitney U test; Significance level at p value < 0.05

**Fig. 2:**
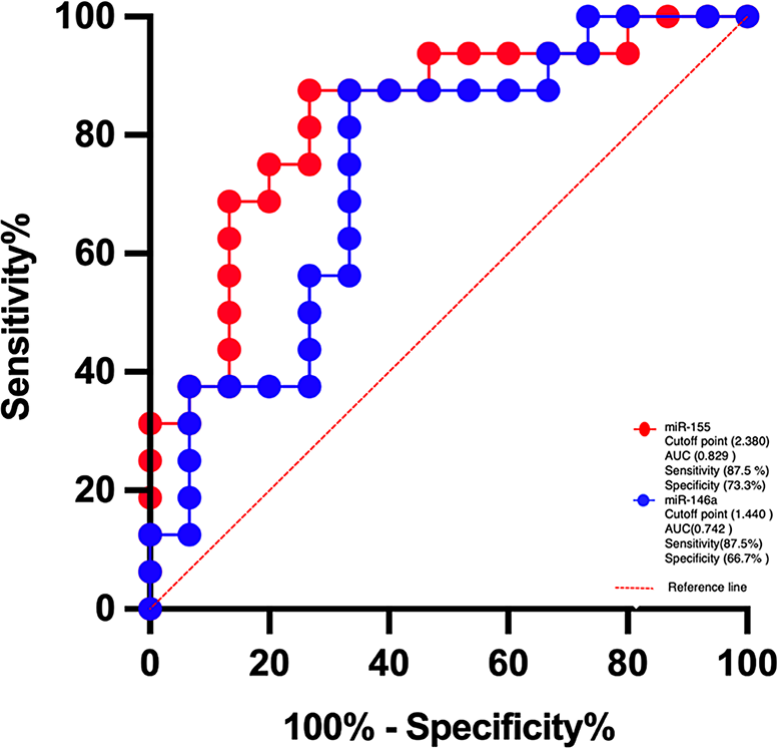
ROC of fold changes (2^−∆∆CT^) of miRNAs for potential differentiating healthy from diseased tissues

The cut off value of fold change of miRNA-155 to differentiate between healthy and diseased tissues was 2.382 (0.829 area under curve, 87.5% sensitivity and 73.3% specificity). For miRNA-146a, the cut off value of fold change was 1.438 (0.742 area under curve, 87.5% sensitivity and 73.3% specificity) as shown in Fig. 2; Table 4.

**Table 4:** Cutoff points of miRNA-155 and miRNA-146a Fold changes to differentiate between healthy and diseased tissue

	Cutoff point	Area under curve	Std. Error	*p* value	Sensitivity %	Specificity %
**miRNA-155**	2.380	0.829	0.076	< 0.05	87.5	73.3
**miRNA-146a**	1.440	0.742	0.092	< 0.05	87.5	66.7

In the case group, the mean level of miRNA-155 was found to be moderately associated with miRNA-146a level (*r* = 0.544, *p* = 0.029). While, no associations between the miRNAs and other study variables were detected as shown in Table 5.

**Table 5:** Spearman’s correlation between miRNAs and case group variables

	miRNA-155		miRNA-146a	
	Correlation coefficient	*P*-value	Correlation coefficient	*P*-value
miRNA-146a	0.544	0.029*	1	-
Age	-0.126	0.642	0.267	0.318
Sex	0.287	0.281	-0.014	0.96
BOP	0.378	0.149	-0.019	0.945
Suppuration	0.058	0.831	-0.292	0.272
Bone loss	-0.059	0.828	-0.136	0.616
PDD	0.076	0.778	-0.007	0.978
GW	0.171	0.526	0.252	0.346
GT	-0.037	0.892	0.085	0.756

* Correlation is significant at the 0.05 level (2-tailed)

## Discussion

To date, this is the first study which assessed the levels of miRNA-155 and miRNA-146a genes in human diseased-tissue of patients with peri-implantitis. In fact, miRNAs have a regulatory role during the process of inflammation. They degrade or suppress messenger RNA molecules, and influence gene expression. Hence, they play a crucial role in several biological and physiological processes such as cell proliferation, growth, differentiation, and apoptosis [50, 51]. There is increasing evidence that dysregulation of miRNAs could contribute to various human diseases such as cancers, cardiovascular disease, chronic hepatitis, and diabetes [52–55]. As peri-implantitis is a bacterial induced inflammatory condition [1], therefore it was expected that the levels of both miRNA-155 and miRNA-146a in diseased tissue would be different compared to healthy peri-implant tissue.

In this study, the levels of both miRNA-155 and miRNA-146a were found to be increased in peri-implantitis affected tissue than healthy peer. This finding is not surprising as many studies have reported that miRNA-155 and miRNA-146a are endotoxin-responsive genes [31, 56, 57]. Peri-implantitis is caused by bacterial biofilm accumulated onto implant surfaces. Bacterial components from the accumulated biofilm such as lipopolysaccharides could induce over-expression of these miRNAs in the affected tissues [56, 57]. TLR-4 on inflammatory cells could recognize bacterial components and thereby trigger the high expression of miRNA-146a within tissues [31]. With time during inflammation, miRNA-146a has been reported to be persist in high levels and acts as a key negative regulator of the immune response. MiRNA-146a represses IL-1 receptor-associated kinase 1 and TNF receptor-associated factor 6 protein mRNAs and thus controlling TLR induced proinflammatory cytokine signaling [31]. On the other hand, limited amount of bacterial accumulation is associated with healthy peri-implant tissue [58].Therefore, no induction for miRNAs overproduction is assumed.

In literature, the expression of miR-155 and miR146a have been extensively studied in periodontitis, dissimilar to peri-implantitis, affected tissue [29, 33–35]. It is worth to mention that this is the first study investigating the level of miRNA-155 in peri-implantitis. While, there is only one animal study which assessed the level of miRNA 146a in ligature-induced peri-implantitis among canine dogs [14]. This study has reported a contrary outcome as the previous study found that miRNA-146a was down-regulated in peri-implantitis. However, the present study assessed the level of miRNAs in human, not animal, tissue which might not necessarily exhibit the same molecular events occurring during the process of peri-implantitis lesion in animals. Moreover, peri-implantitis lesion was induced in the previous animal study, whereas this study involved volunteers already having peri-implantitis. Furthermore, the previous study used a split-mouth design, while this study investigated sample distributed in parallel groups with possibly less controlling of inter-group confounders. Thus, these, above-mentioned, differences in studied samples might explain the inconsistence in the findings. Therefore, more studies are highly suggested to resolve this disagreement regarding this finding.

MiRNA-155 is an important regulator of TLR-related disorders. The stimulation of cells with bacterial LPS increased the expression of miRNA-155, which worked on NF-B via negative feedback, regulating the release of numerous cytokines and influencing the inflammatory response [31, 59]. Because both miRNA-155 and miRNA-146a have a role in LPS-induced macrophage activation, these variables are frequently examined together. Their expression profiles, however, differed in various tissues. In periodontitis patients, for example, miRNA-146a was found to be overexpressed in inflammatory gingival tissue, while miRNA-155 was downregulated in some reports [34, 60] and upregulated in others [29, 33]. However, it has been reported that the expression of miRNA-155 and miRNA-146a in the GCF of patients with periodontitis was upregulated [33]. No doubt, miRNA-146a and miRNA-155 function in a two-tiered manner. During the initiation of inflammation, miRNA-146a is activated in response to low LPS doses, while miRNA-155 is gradually elevated to full expression and acts as a limiter of proinflammatory gene expression once the miRNA-146a-dependent anti-inflammatory impact is lost [33, 61].

Both miRNA-155 and miRNA-146a expressions showed a considerable discriminating efficacy to differentiate peri-implantitis affected from healthy tissues with accepted levels of sensitivity and specificity. For the diagnosis of periodontitis, similar findings have been reported that the level of both miRNA-146a and miRNA-155 could differentiate between healthy and periodontitis patients with high accuracy based on ROC curves analysis [33]. This, in turn, highlights the possibility of using these miRNAs as potential biomarkers to predict peri-implantitis. MiRNA-146 and miRNA-155 are vital in controlling the immune response and inflammation associated with periodontal disease. Their consistent presence in bodily fluids makes them very promising as diagnostic biomarkers [62]. At the same time, their ability to regulate biological processes presents prospective opportunities for targeting and controlling peri-implantitis and enhancing periodontal health. More research is required to convert these discoveries into practical uses in the medical field, specifically establishing uniform diagnostic standards and improving the methods of administering miRNA-based treatments [35]. In fact, it is not reasonable to use the expression of these miRNAs in tissue for the diagnosis of peri-implant disease. More clinically relevant, it is suggested that the levels of these biomarker in peri-implant gingival fluid might have the same discriminating efficacy. To approve this suggestion, future studies are highly suggested.

The present study showed nonsignificant association between miRNA-146a and mi-RNA-155 levels with bone loss and pocket depth. This might reflect the roles of these miRNAs in regulating the immunological response or maintaining immunological hemostasis in peri-implantitis disease.

As a limitation in this study, plaque index was not recorded. However, BOP was recorded in both groups which provided more objective indicator for the health status of peri-implant tissue. Moreover, it was better to use a split-mouth design in this study. This design provides better control of unexpected inter-group confounding factors. However, strict inclusion and exclusion criteria were used in this study in order to minimize the effects of unexpected inter-group confounders. Furthermore, the unrevealed association between the miRNAs expression and clinical parameters might be attributed to the small sample size considered in this study. However, the sample size was estimated to detect the difference of miRNA-155 expression (as the primary outcome) between the study groups. Future studies with larger sample size are suggested to resolve this issue.

## Conclusion

This study demonstrates that the expressions of miRNA-146a and miRNA-155 are different between healthy and peri-implantitis affected tissues. Both miRNAs might potentially able to discriminate the healthy from peri-implantitis affected tissues. Further studies are suggested to investigate the role of these miRNAs within the course of peri-implant tissue inflammation.

### Electronic supplementary material

Below is the link to the electronic supplementary material.

Supplementary Material 1

Supplementary Material 2

Supplementary Material 3

Supplementary Material 4

## Data Availability

The datasets supporting the conclusions of this article are included within the article and its additional files.

## Abbreviations

(miRNAs): microRNAs
(TLRs): Toll-like receptors
(PPD): probing pocket depth
(BOP): bleeding on probing
(qPCR): polymerase chain reaction
