# Supplementary material for: Expression of miRNAs (146a and 155) in human peri-implant tissue affected by peri-implantitis: a case control study
Source: BMC Oral Health. 2024 Jul 28;24:856. doi: 10.1186/s12903-024-04579-x (PMC11283691; doi:10.1186/s12903-024-04579-x)
Supplement: Supplementary file 1 — Supplementary Material 1 [file 12903_2024_4579_MOESM1_ESM.pdf]

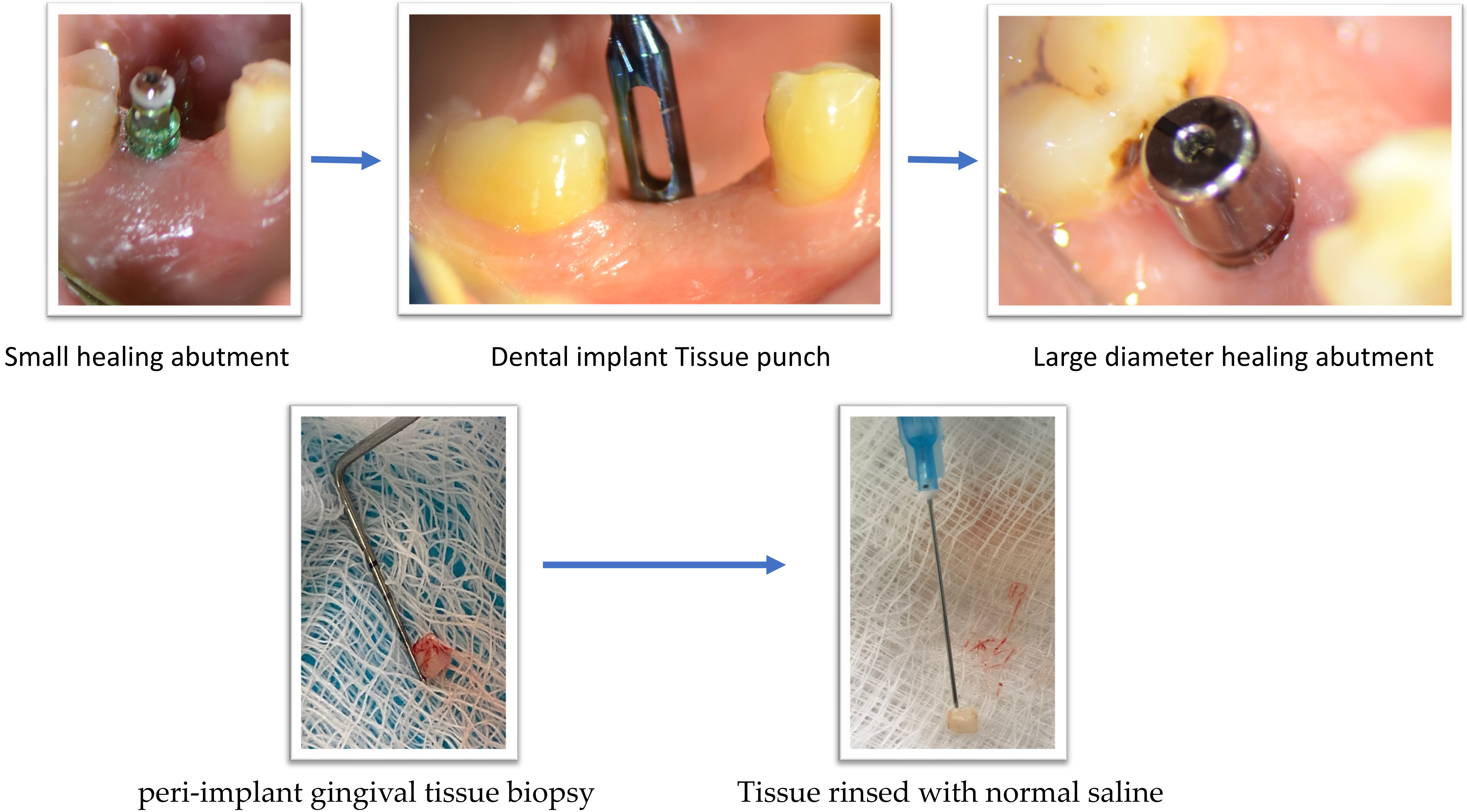

Figure S1.peri-implant tissue sampling of the control group
