# Supplementary figures and images for: Expression of miRNAs (146a and 155) in human peri-implant tissue affected by peri-implantitis: a case control study
Source: BMC Oral Health. 2024 Jul 28;24:856. doi: 10.1186/s12903-024-04579-x (PMC11283691; doi:10.1186/s12903-024-04579-x)

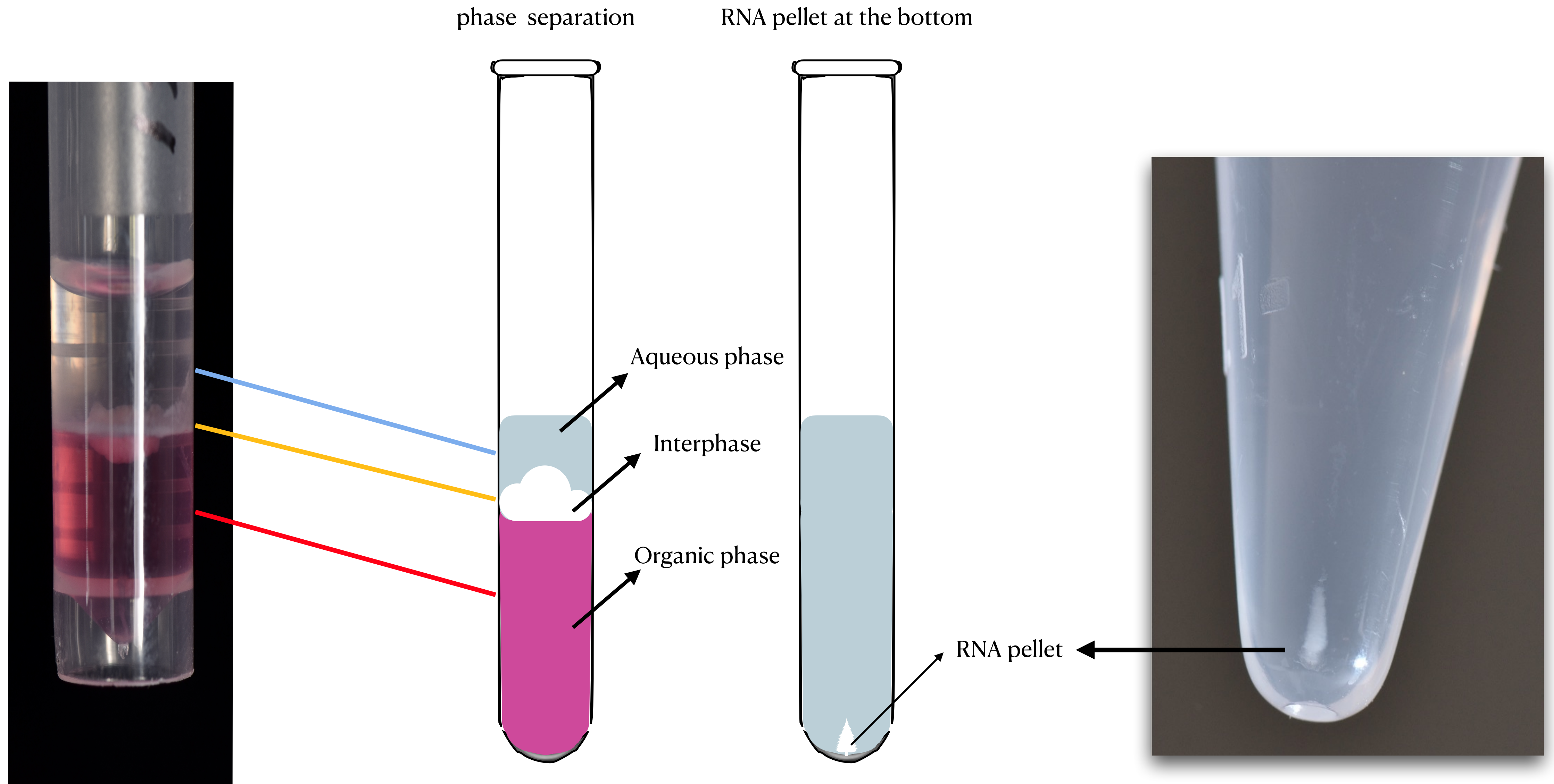

**Finger S2.** RNA Extraction showed the Tri-phases and RNA pullet

Supplement: Supplementary file 2 — Supplementary Material 2 [file 12903_2024_4579_MOESM2_ESM.pdf]
