# Supplementary material for: Expression of miRNAs (146a and 155) in human peri-implant tissue affected by peri-implantitis: a case control study
Source: BMC Oral Health. 2024 Jul 28;24:856. doi: 10.1186/s12903-024-04579-x (PMC11283691; doi:10.1186/s12903-024-04579-x)
Supplement: Supplementary file 3 — Supplementary Material 3 [file 12903_2024_4579_MOESM3_ESM.pdf]

Table S1. Basic variables of the study groups

group	dct	ddct	miR-155	dct	ddct	miR-146	BOP	Suppurtion	PDD	Bone loss
1	**0.22**	**-1.28**	2.42	-2.14	-2.14	2.22	0.8	0	5.5	3.5
1	**-3.20**	**-4.70**	25.97	-5.64	-5.64	25.16	1	0	5.5	3.2
1	**-4.04**	**-5.54**	46.63	-6.46	-6.46	44.46	1	0	5.5	3.1
1	**-2.74**	**-4.24**	18.92	-4.50	-4.50	11.41	1	0.3	5.3	4
1	**-0.92**	**-2.42**	5.36	-2.13	-2.13	4.25	0.6	0	4.8	3
1	**1.00**	**-0.50**	1.41	-2.95	-2.95	3.90	0.6	0	5.6	3.6
1	**3.78**	**2.28**	0.21	-3.07	-3.07	2.21	o.8	0	5.3	3.5
1	**-0.23**	**-1.73**	3.32	-2.36	-2.36	2.59	0.8	0	4.7	3
1	**0.04**	**-1.47**	2.76	-2.96	-2.96	3.94	1	0.5	5.6	4.3
1	**-1.84**	**-3.34**	10.11	-4.27	-4.27	9.76	0.6	0	6.3	3.8
1	**-3.37**	**-4.87**	29.28	-4.45	-4.45	11.04	0.8	0	7.5	4.7
1	**-3.60**	**-5.10**	34.24	-1.73	-1.73	1.68	1	0.5	5.3	3.25
1	**-0.93**	**-2.43**	5.39	0.51	0.51	0.36	1	0	6.1	4
1	**-0.36**	**-1.86**	3.62	1.00	1.00	0.25	1	0.6	6.3	5.25
1	**-1.07**	**-2.57**	5.93	-4.37	-4.37	10.48	0.8	0	5.5	3.5
1	**-0.51**	**-2.01**	4.04	-2.68	-2.68	3.23	0.6	0	5.5	1.8
2	-0.30	-1.80	3.48	-3.91	-3.91	7.59	0	0	2.5	0
2	-2.21	-3.71	13.11	-4.85	-4.85	14.54	0	0	2	0
2	-0.18	-1.68	3.20	-3.98	-3.98	7.97	0	0	2	0
2	1.59	0.09	0.94	-3.74	-3.74	6.74	0	0	1	0
2	0.82	-0.68	1.61	0.70	0.70	0.31	0	0	1.5	0
2	-1.16	-2.66	6.32	-1.12	-1.12	1.10	0	0	1.4	0
2	4.28	2.78	0.15	1.96	1.96	0.13	0	0	2	0
2	2.83	1.33	0.40	0.14	0.14	0.46	0	0	1.9	0
2	0.33	-1.17	2.24	-2.69	-2.69	3.26	0	0	1.7	0
2	3.79	2.29	0.20	1.22	1.22	0.22	0	0	1.7	0
2	2.92	1.42	0.37	-1.24	-1.24	1.20	0	0	2.5	0
2	5.50	4.00	0.06	2.02	2.02	0.12	0	0	1.8	0
2	2.39	0.89	0.54	-0.22	-0.22	0.59	0	0	2	0
2	0.27	-1.23	2.34	-0.96	-0.96	0.98	0	0	2.5	0
2	1.64	0.14	0.91	1.92	1.92	0.13	0	0	2.1	0

Dct = (ΔCT = CT gene - CT Housekeeping gene.)
Ddct = (ΔΔCT = ΔCT Treated or Control – Average ΔCT Control.)
miR-155 = (Fold change = "2-ΔΔCT")
miR-146a = (Fold change = "2-ΔΔCT")
BOP = (Bleeding on probing.)
PDD = (Peri-implant pocket depth.)
