# Supplementary material for: Expression of miRNAs (146a and 155) in human peri-implant tissue affected by peri-implantitis: a case control study
Source: BMC Oral Health. 2024 Jul 28;24:856. doi: 10.1186/s12903-024-04579-x (PMC11283691; doi:10.1186/s12903-024-04579-x)
Supplement: Supplementary file 4 — Supplementary Material 4 [file 12903_2024_4579_MOESM4_ESM.pdf]

```
 NEW FILE.
DATASET NAME DataSet1 WINDOW=FRONT.
SORT CASES  BY group.
SPLIT FILE SEPARATE BY group.
DESCRIPTIVES VARIABLES=dct155 ddct155 foldmiR155 dct146 ddct146 folddmiR146 Bo
p suppuration PPD
    boneloss GW GTT
  /STATISTICS=MEAN STDDEV SKEWNESS.
```

```
 DATASET ACTIVATE DataSet1.
DATASET CLOSE DataSet0.
DESCRIPTIVES VARIABLES=dct155 ddct155 foldmiR155 dct146 ddct146 folddmiR146 Bo
p suppuration PPD
    boneloss GW GTT
  /STATISTICS=MEAN STDDEV SKEWNESS.
```

Descriptives

Notes

Output Created		15-APR-2024 16:46:...
Comments		
Input	Active Dataset	DataSet1
	Filter	<none>
	Weight	<none>
	Split File	group
	N of Rows in Working Data File	31
Missing Value Handling	Definition of Missing	User defined missing values are treated as missing.
	Cases Used	All non-missing data are used.
Syntax		DESCRIPTIVES VARIABLES=dct155 ddct155 foldmiR155 dct146 ddct146 folddmiR146 Bop suppuration PPD boneloss GW GTT /STATISTICS=MEAN STDDEV SKEWNESS.
Resources	Processor Time	00:00:00.00
	Elapsed Time	00:00:00.00

group = 1

Descriptive Statistics[a]

	N	Mean	Std. Deviation	Skewness	
	Statistic	Statistic	Statistic	Statistic	Std. Error
dct155	16	-1.1106	2.01119	.615	.564
ddct155	16	-2.6112	2.01081	.616	.564
foldmiR155	16	12.4756	14.13580	1.333	.564
dct146	16	-3.0125	1.97875	.415	.564
ddct146	16	-3.0125	1.97875	.415	.564
folddmiR146	16	8.5588	11.47055	2.438	.564
Bop	15	.8400	.17238	-.433	.580
suppuration	16	.1187	.21975	1.494	.564
PPD	16	5.6438	.66229	1.437	.564
boneloss	16	3.5938	.78759	-.003	.564
GW	16	5.6313	1.99991	.792	.564
GTT	16	1.8781	.40206	-.496	.564
Valid N (listwise)	15				

a. group = 1

group = 2

Descriptive Statistics[a]

	N	Mean	Std. Deviation	Skewness	
	Statistic	Statistic	Statistic	Statistic	Std. Error
dct155	15	1.5007	2.13515	.161	.580
ddct155	15	.0007	2.13515	.161	.580
foldmiR155	15	2.3913	3.42094	2.533	.580
dct146	15	-.9833	2.37154	-.255	.580
ddct146	15	-.9833	2.37154	-.255	.580
folddmiR146	15	3.0227	4.27544	1.717	.580
Bop	15	.0000	.00000	.	.
suppuration	15	.0000	.00000	.	.
PPD	15	1.9067	.41998	-.361	.580
boneloss	15	.0000	.00000	.	.
GW	15	3.9733	.39364	.983	.580
GTT	15	1.8533	.42404	-.209	.580
Valid N (listwise)	15				

a. group = 2

```
 SPLIT FILE OFF.
T-TEST GROUPS=group(1 2)
  /MISSING=ANALYSIS
  /VARIABLES=dct155 ddct155 foldmiR155 dct146 ddct146 folddmiR146
  /CRITERIA=CI(.95).
```

T-Test

Notes

Output Created		15-APR-2024 16:48:...
Comments		
Input	Active Dataset	DataSet1
	Filter	<none>
	Weight	<none>
	Split File	<none>
	N of Rows in Working Data File	31
Missing Value Handling	Definition of Missing	User defined missing values are treated as missing.
	Cases Used	Statistics for each analysis are based on the cases with no missing or out-of-range data for any variable in the analysis.
Syntax		T-TEST GROUPS=group (1 2)<br>/MISSING=ANALYSIS<br>/VARIABLES=dct155 ddct155 foldmiR155 dct146 ddct146 folddmiR146<br>/CRITERIA=CI(.95).
Resources	Processor Time	00:00:00.00
	Elapsed Time	00:00:00.00

Group Statistics

	group	N	Mean	Std. Deviation	Std. Error Mean
dct155	1	16	-1.1106	2.01119	.50280
	2	15	1.5007	2.13515	.55129
ddct155	1	16	-2.6112	2.01081	.50270
	2	15	.0007	2.13515	.55129
foldmiR155	1	16	12.4756	14.13580	3.53395
	2	15	2.3913	3.42094	.88328
dct146	1	16	-3.0125	1.97875	.49469
	2	15	-.9833	2.37154	.61233
ddct146	1	16	-3.0125	1.97875	.49469
	2	15	-.9833	2.37154	.61233
folddmiR146	1	16	8.5588	11.47055	2.86764
	2	15	3.0227	4.27544	1.10391

Independent Samples Test

		Levene's Test for Equality of Variances		t-test for Equality of Means	
		F	Sig.	t	df
dct155	Equal variances assumed	.227	.637	-3.507	29
	Equal variances not assumed			-3.500	28.544
ddct155	Equal variances assumed	.228	.637	-3.508	29
	Equal variances not assumed			-3.501	28.543
foldmiR155	Equal variances assumed	20.965	.000	2.687	29
	Equal variances not assumed			2.768	16.862
dct146	Equal variances assumed	1.185	.285	-2.593	29
	Equal variances not assumed			-2.578	27.360
ddct146	Equal variances assumed	1.185	.285	-2.593	29
	Equal variances not assumed			-2.578	27.360
folddmiR146	Equal variances assumed	3.640	.066	1.757	29
	Equal variances not assumed			1.802	19.321

Independent Samples Test

		t-test for Equality of Means		
		Sig. (2-tailed)	Mean Difference	Std. Error Difference
dct155	Equal variances assumed	.001	-2.61129	.74466
	Equal variances not assumed	.002	-2.61129	.74614
ddct155	Equal variances assumed	.001	-2.61192	.74459
	Equal variances not assumed	.002	-2.61192	.74608
foldmiR155	Equal variances assumed	.012	10.08429	3.75231
	Equal variances not assumed	.013	10.08429	3.64266
dct146	Equal variances assumed	.015	-2.02917	.78249
	Equal variances not assumed	.016	-2.02917	.78719
ddct146	Equal variances assumed	.015	-2.02917	.78249
	Equal variances not assumed	.016	-2.02917	.78719
folddmiR146	Equal variances assumed	.090	5.53608	3.15124
	Equal variances not assumed	.087	5.53608	3.07278

Independent Samples Test

		t-test for Equality of Means	
		95% Confidence Interval of the Difference	
		Lower	Upper
dct155	Equal variances assumed	-4.13428	-1.08830
	Equal variances not assumed	-4.13838	-1.08420
ddct155	Equal variances assumed	-4.13477	-1.08906
	Equal variances not assumed	-4.13888	-1.08495
foldmiR155	Equal variances assumed	2.40995	17.75863
	Equal variances not assumed	2.39416	17.77443
dct146	Equal variances assumed	-3.62955	-.42879
	Equal variances not assumed	-3.64334	-.41499
ddct146	Equal variances assumed	-3.62955	-.42879
	Equal variances not assumed	-3.64334	-.41499
folddmiR146	Equal variances assumed	-.90892	11.98109
	Equal variances not assumed	-.88810	11.96027

```
 *Nonparametric Tests: Independent Samples.
NPTESTS
  /INDEPENDENT TEST (dct155 ddct155 foldmiR155 dct146 ddct146 folddmiR146) GRO
UP (group) MANN_WHITNEY
  /MISSING SCOPE=ANALYSIS USERMISSING=EXCLUDE
  /CRITERIA ALPHA=0.05  CILEVEL=95.
```

Nonparametric Tests

Notes

Output Created		15-APR-2024 16:49:...
Comments		
Input	Active Dataset	DataSet1
	Filter	<none>
	Weight	<none>
	Split File	<none>
	N of Rows in Working Data File	31
Syntax		NPTESTS /INDEPENDENT TEST (dct155 ddct155 foldmiR155 dct146 ddct146 folddmiR146) GROUP (group) MANN_WHITNEY /MISSING SCOPE=ANALYSIS USERMISSING=EXCLUDE /CRITERIA ALPHA=0.05 CILEVEL=95.
Resources	Processor Time	00:00:01.58
	Elapsed Time	00:00:02.00

Hypothesis Test Summary

	Null Hypothesis	Test	Sig.
1	The distribution of dct155 is the same across categories of group.	Independent-Samples Mann-Whitney U Test	.001[a]
2	The distribution of ddct155 is the same across categories of group.	Independent-Samples Mann-Whitney U Test	.001[a]
3	The distribution of foldmiR155 is the same across categories of group.	Independent-Samples Mann-Whitney U Test	.001[a]
4	The distribution of dct146 is the same across categories of group.	Independent-Samples Mann-Whitney U Test	.021[a]
5	The distribution of ddct146 is the same across categories of group.	Independent-Samples Mann-Whitney U Test	.021[a]
6	The distribution of folddmiR146 is the same across categories of group.	Independent-Samples Mann-Whitney U Test	.021[a]

Hypothesis Test Summary

	Decision
1	Reject the null hypothesis.
2	Reject the null hypothesis.
3	Reject the null hypothesis.
4	Reject the null hypothesis.
5	Reject the null hypothesis.
6	Reject the null hypothesis.

Asymptotic significances are displayed. The significance level is .050.

a. Exact significance is displayed for this test.

Independent-Samples Mann-Whitney U Test

dct155 across group

Independent-Samples Mann-Whitney U Test Summary

Total N	31
Mann-Whitney U	199.000
Wilcoxon W	319.000
Test Statistic	199.000
Standard Error	25.298
Standardized Test Statistic	3.123
Asymptotic Sig.(2-sided test)	.002
Exact Sig.(2-sided test)	.001

Independent-Samples Mann-Whitney U Test

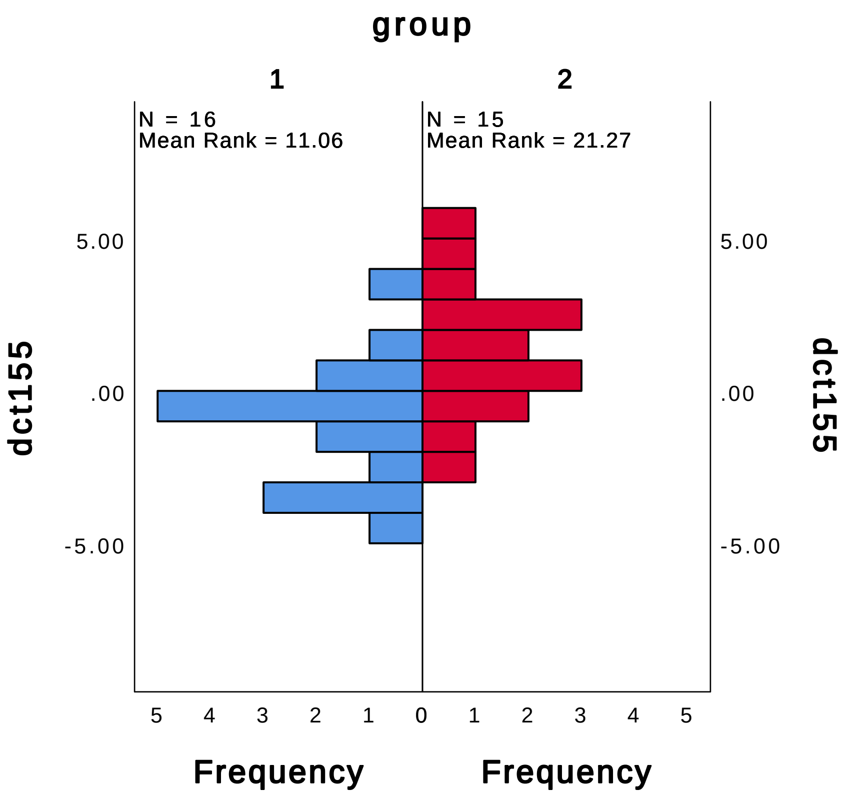

ddct155 across group

Independent-Samples Mann-Whitney U Test Summary

Total N	31
Mann-Whitney U	199.000
Wilcoxon W	319.000
Test Statistic	199.000
Standard Error	25.298
Standardized Test Statistic	3.123
Asymptotic Sig.(2-sided test)	.002
Exact Sig.(2-sided test)	.001

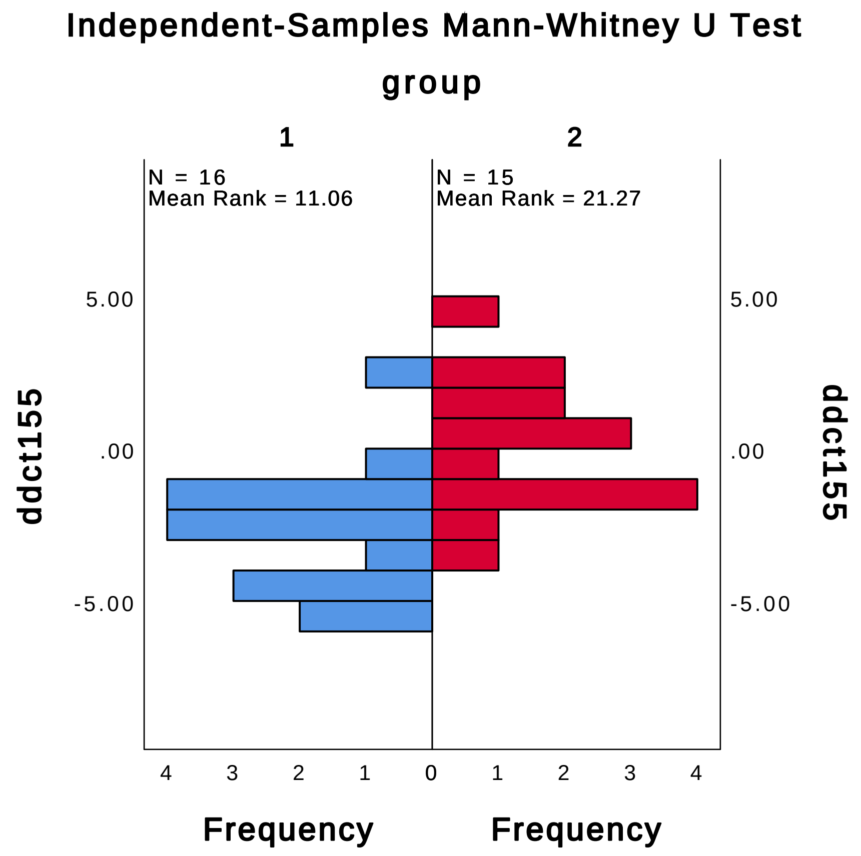

foldmiR155 across group

Independent-Samples Mann-Whitney U Test Summary

Total N	31
Mann-Whitney U	41.000
Wilcoxon W	161.000
Test Statistic	41.000
Standard Error	25.298
Standardized Test Statistic	-3.123
Asymptotic Sig.(2-sided test)	.002
Exact Sig.(2-sided test)	.001

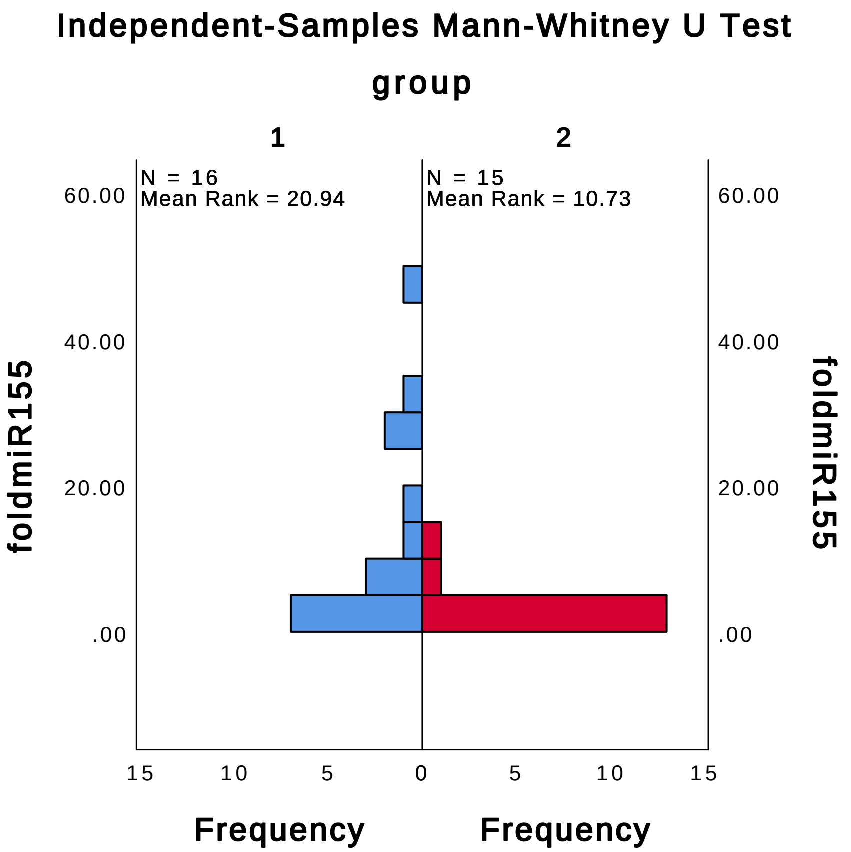

dct146 across group

Independent-Samples Mann-Whitney U Test Summary

Total N	31
Mann-Whitney U	178.000
Wilcoxon W	298.000
Test Statistic	178.000
Standard Error	25.298
Standardized Test Statistic	2.293
Asymptotic Sig.(2-sided test)	.022
Exact Sig.(2-sided test)	.021

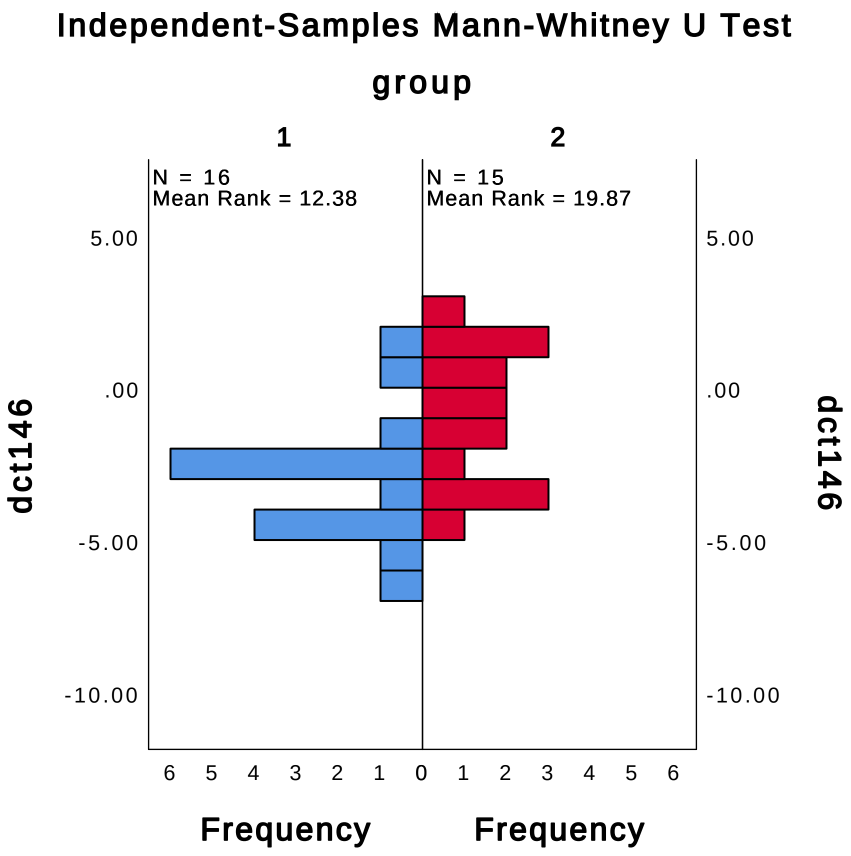

ddct146 across group

Independent-Samples Mann-Whitney U Test Summary

Total N	31
Mann-Whitney U	178.000
Wilcoxon W	298.000
Test Statistic	178.000
Standard Error	25.298
Standardized Test Statistic	2.293
Asymptotic Sig.(2-sided test)	.022
Exact Sig.(2-sided test)	.021

Independent-Samples Mann-Whitney U Test

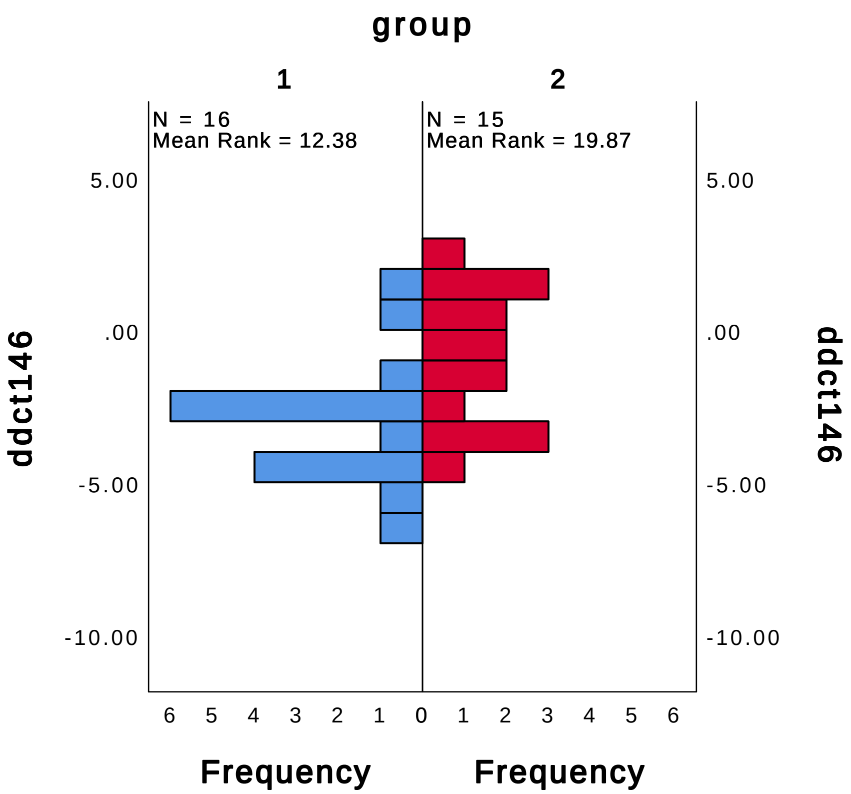

folddmiR146 across group

Independent-Samples Mann-Whitney U Test Summary

Total N	31
Mann-Whitney U	62.000
Wilcoxon W	182.000
Test Statistic	62.000
Standard Error	25.296
Standardized Test Statistic	-2.293
Asymptotic Sig.(2-sided test)	.022
Exact Sig.(2-sided test)	.021

Independent-Samples Mann-Whitney U Test

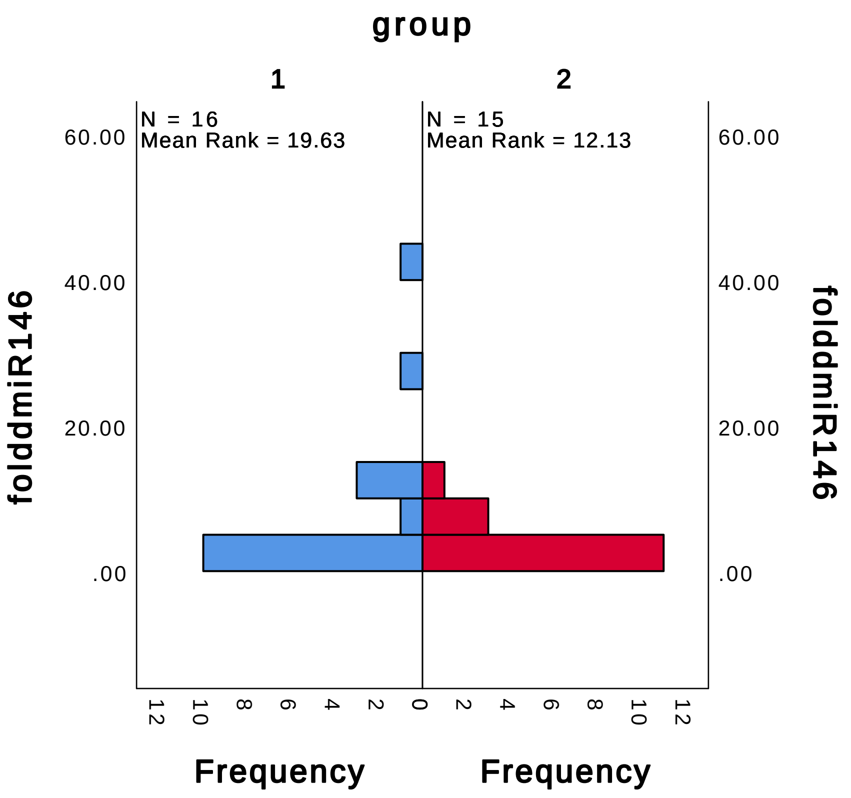

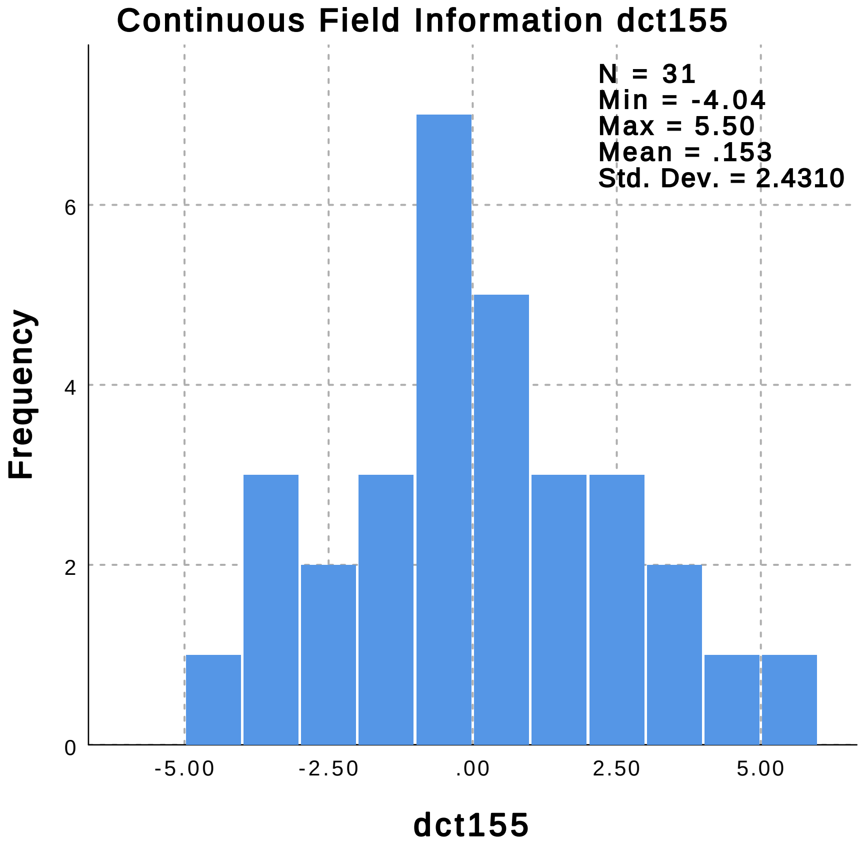

Continuous Field Information dct155
N = 31
Min = -4.04
Max = 5.50
Mean = .153
Std. Dev. = 2.4310
Frequency
0
2
4
6
-5.00
-2.50
.00
2.50
5.00
dct155

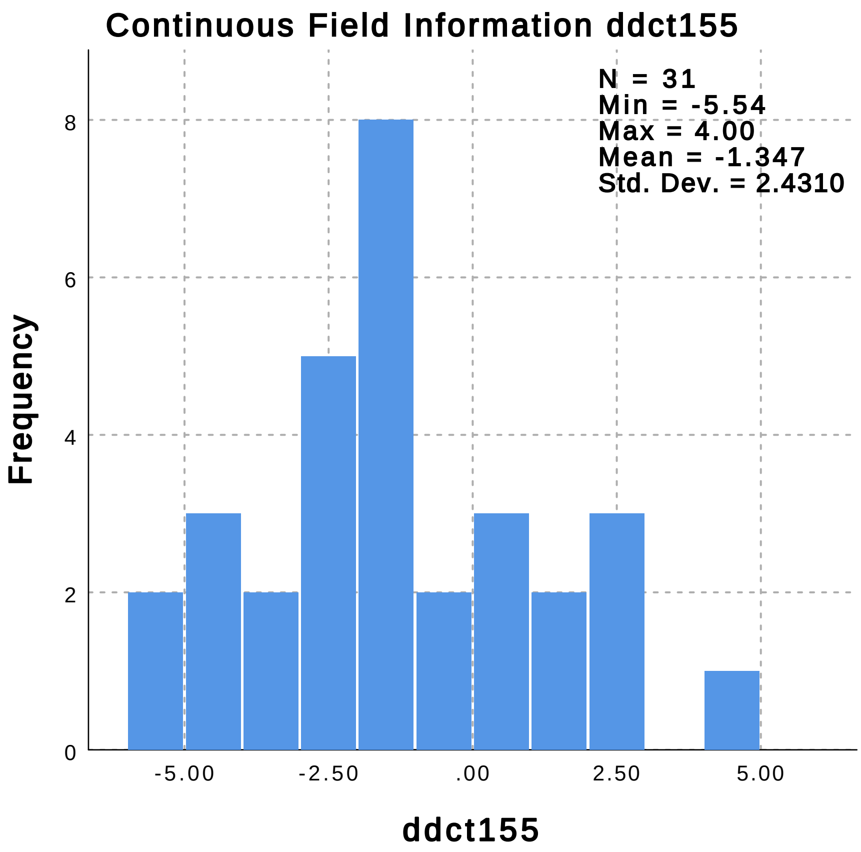
Continuous Field Information ddct155
N = 31
Min = -5.54
Max = 4.00
Mean = -1.347
Std. Dev. = 2.4310
Frequency
0
2
4
6
8
-5.00
-2.50
.00
2.50
5.00
ddct155

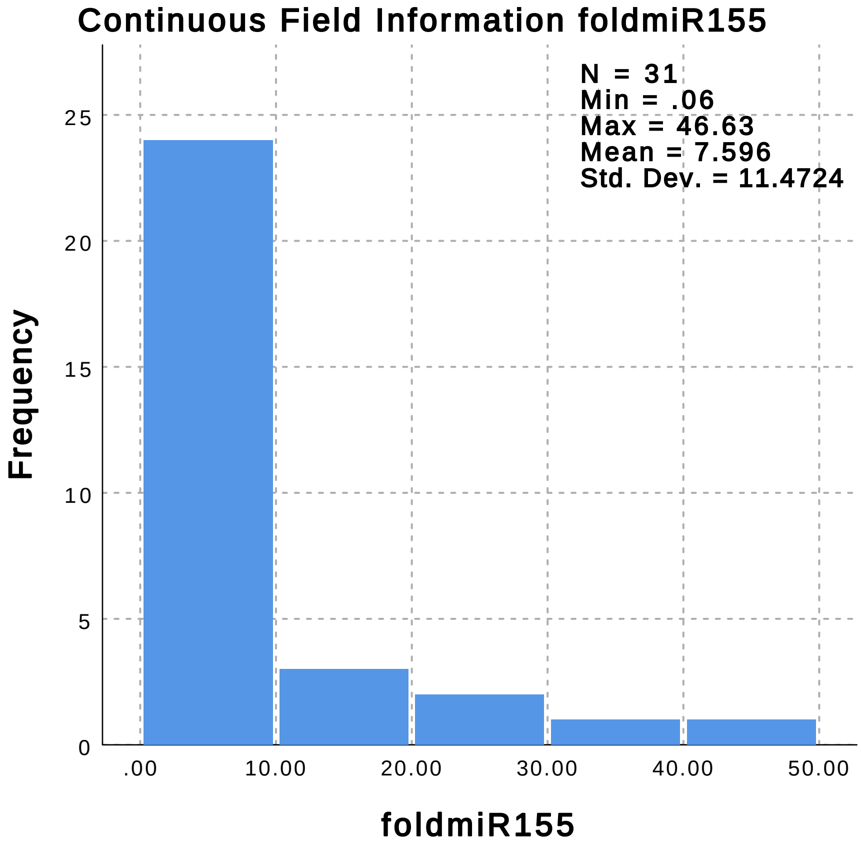
Continuous Field Information foldmiR155
N = 31
Min = .06
Max = 46.63
Mean = 7.596
Std. Dev. = 11.4724
Frequency
0
5
10
15
20
25
.00
10.00
20.00
30.00
40.00
50.00
foldmiR155

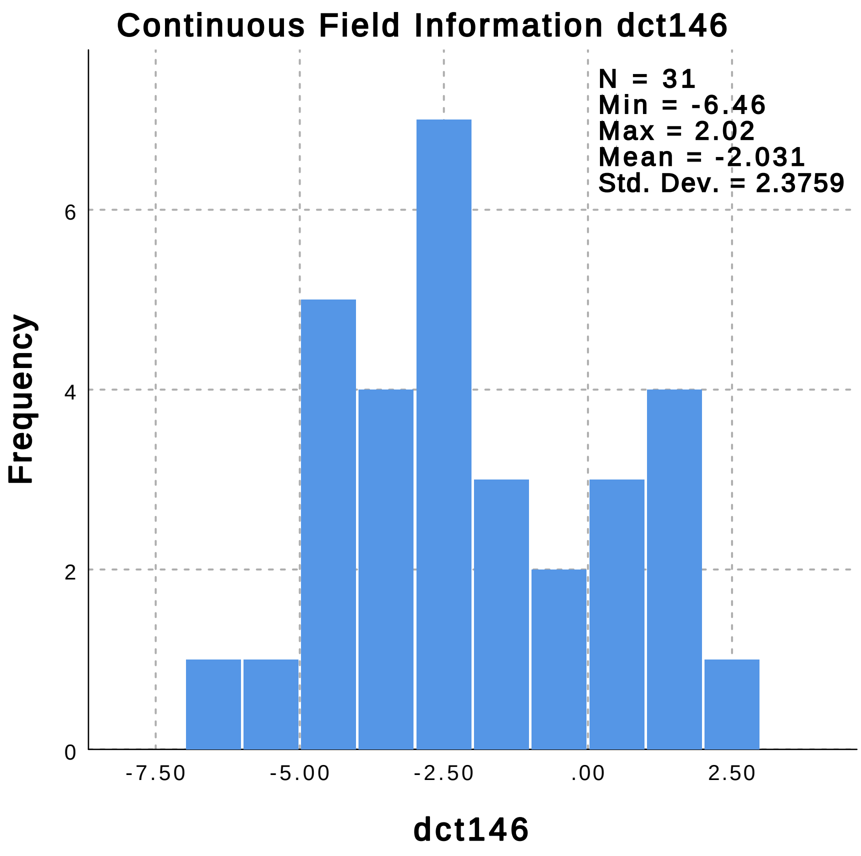
Continuous Field Information dct146
N = 31
Min = -6.46
Max = 2.02
Mean = -2.031
Std. Dev. = 2.3759
Frequency
0
2
4
6
-7.50
-5.00
-2.50
.00
2.50
dct146

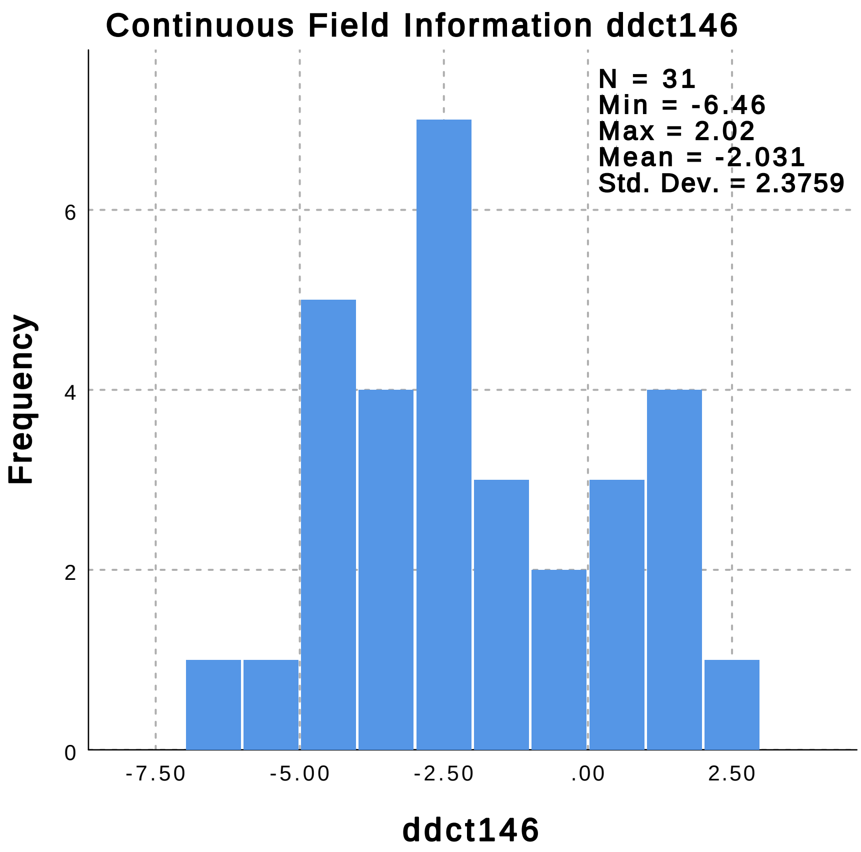
Continuous Field Information ddct146
N = 31
Min = -6.46
Max = 2.02
Mean = -2.031
Std. Dev. = 2.3759
Frequency
0
2
4
6
-7.50
-5.00
-2.50
.00
2.50
ddct146

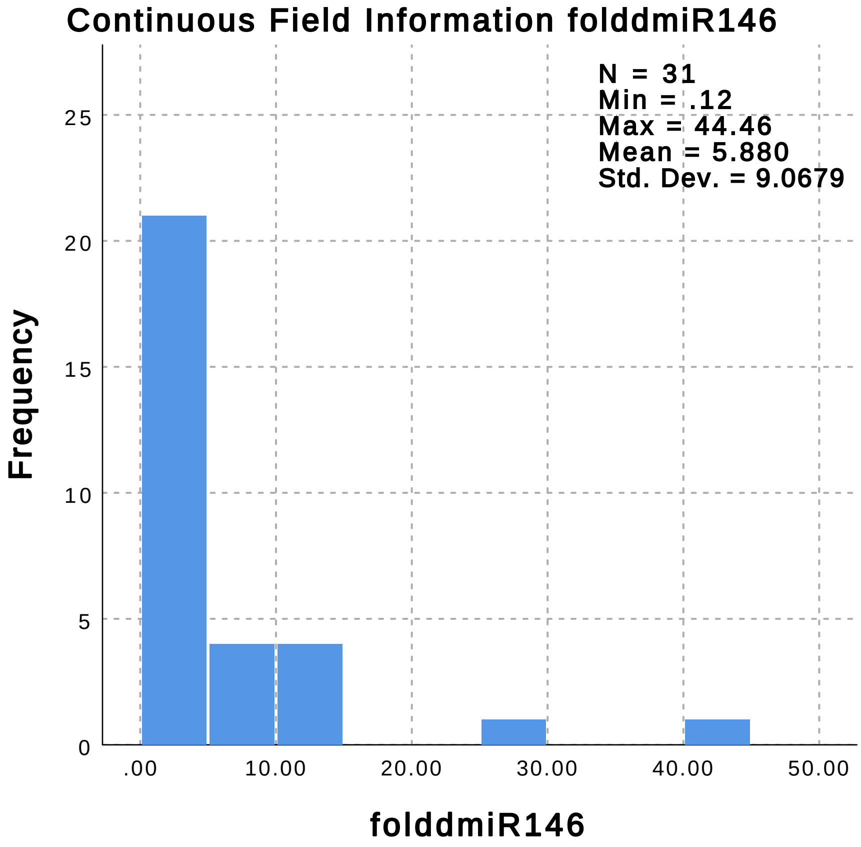
Continuous Field Information folddmiR146
N = 31
Min = .12
Max = 44.46
Mean = 5.880
Std. Dev. = 9.0679
Frequency
0
5
10
15
20
25
.00
10.00
20.00
30.00
40.00
50.00
folddmiR146

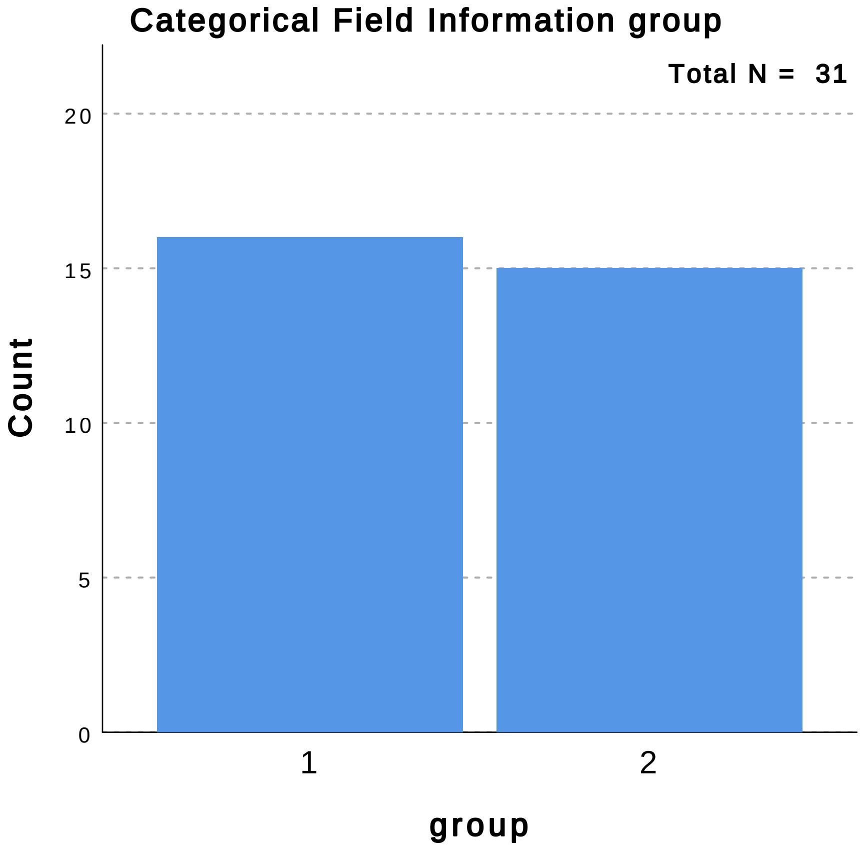


```
ROC foldmiR155 folddmiR146 BY group (1)
 /PLOT=CURVE(REFERENCE)
 /PRINT=SE COORDINATES
 /CRITERIA=CUTOFF(INCLUDE) TESTPOS(LARGE) DISTRIBUTION(FREE) CI(95)
 /MISSING=EXCLUDE.
```

ROC Curve

Notes

Output Created		15-APR-2024 16:53:...
Comments		
Input	Active Dataset	DataSet1
	Filter	<none>
	Weight	<none>
	Split File	<none>
	N of Rows in Working Data File	31
Missing Value Handling	Definition of Missing	User-defined missing values are treated as missing.
	Cases Used	Statistics are based on all cases with valid data for all variables in the analysis.
Syntax		ROC foldmiR155 folddmiR146 BY group (1) /PLOT=CURVE (REFERENCE) /PRINT=SE COORDINATES /CRITERIA=CUTOFF (INCLUDE) TESTPOS (LARGE) DISTRIBUTION (FREE) CI(95) /MISSING=EXCLUDE.
Resources	Processor Time	00:00:00.61
	Elapsed Time	00:00:01.00

Case Processing Summary

group	Valid N (listwise)
Positive[a]	16
Negative	15

Larger values of the test result variable(s) indicate stronger evidence for a positive actual state.

a. The positive actual state is 1.

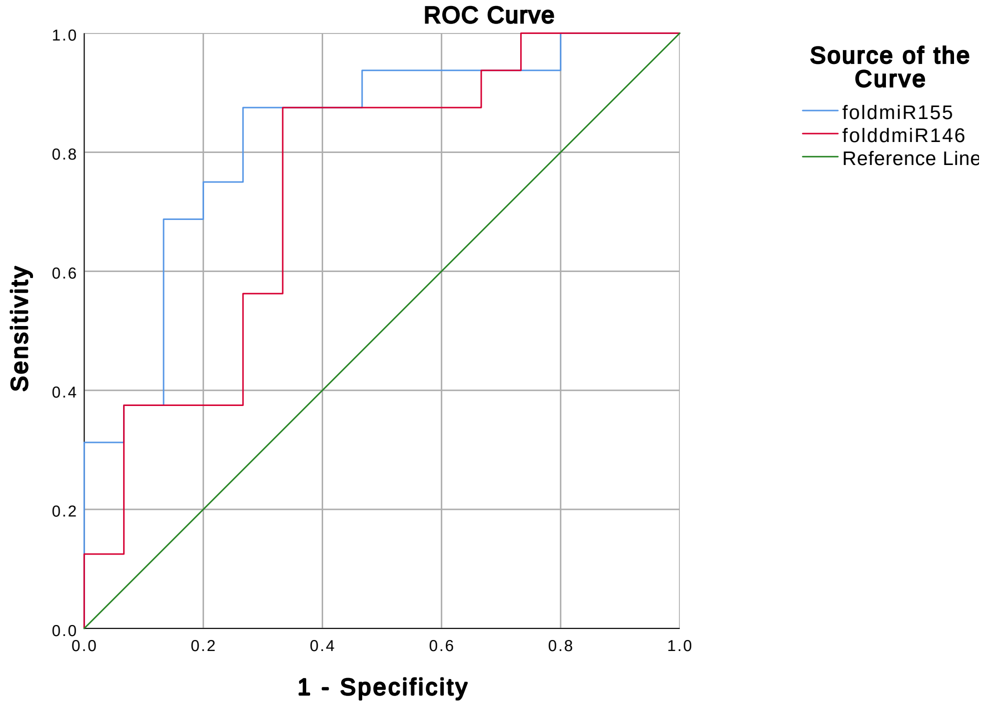

Area Under the Curve

Test Result Variable(s)	Area	Std. Error[a]	Asymptotic Sig.[b]	Asymptotic 95% Confidence Interval	
				Lower Bound	Upper Bound
foldmiR155	.829	.076	.002	.681	.978
folddmiR146	.742	.092	.022	.562	.921

a. Under the nonparametric assumption

b. Null hypothesis: true area = 0.5

Coordinates of the Curve

Test Result Variable(s)	Positive if Greater Than or Equal To[a]	Sensitivity	1 - Specificity
foldmiR155	-.9400	1.000	1.000
	.1050	1.000	.933
	.1750	1.000	.867
	.2050	1.000	.800
	.2900	.938	.800
	.3850	.938	.733
	.4700	.938	.667
	.7250	.938	.600
	.9250	.938	.533
	1.1750	.938	.467
	1.5100	.875	.467
	1.9250	.875	.400
	2.2900	.875	.333
	2.3800	.875	.267
	2.5900	.813	.267
	2.9800	.750	.267
	3.2600	.750	.200
	3.4000	.688	.200
	3.5500	.688	.133
	3.8300	.625	.133
	4.7000	.563	.133
	5.3750	.500	.133
	5.6600	.438	.133
	6.1250	.375	.133
	8.2150	.375	.067
	11.6100	.313	.067
	16.0150	.313	.000
	22.4450	.250	.000
	27.6250	.188	.000
	31.7600	.125	.000
	40.4350	.063	.000
	47.6300	.000	.000
folddmiR146	-.8800	1.000	1.000
	.1250	1.000	.933
	.1750	1.000	.800

Coordinates of the Curve

Test Result Variable(s)	Positive if Greater Than or Equal To[a]	Sensitivity	1 - Specificity
	.2350	1.000	.733
	.2800	.938	.733
	.3350	.938	.667
	.4100	.875	.667
	.5250	.875	.600
	.7850	.875	.533
	1.0400	.875	.467
	1.1500	.875	.400
	1.4400	.875	.333
	1.9450	.813	.333
	2.2150	.750	.333
	2.4050	.688	.333
	2.9100	.625	.333
	3.2450	.563	.333
	3.5800	.563	.267
	3.9200	.500	.267
	4.0950	.438	.267
	5.4950	.375	.267
	7.1650	.375	.200
	7.7800	.375	.133
	8.8650	.375	.067
	10.1200	.313	.067
	10.7600	.250	.067
	11.2250	.188	.067
	12.9750	.125	.067
	19.8500	.125	.000
	34.8100	.063	.000
	45.4600	.000	.000

a. The smallest cutoff value is the minimum observed test value minus 1, and the largest cutoff value is the maximum observed test value plus 1. All the other cutoff values are the averages of two consecutive ordered observed test values.

```
SAVE OUTFILE='/Volumes/Extreme SSD/new for review 2022-A/paper one/data paper
one /paper one '+
    'data.sav'
  /COMPRESSED.
NONPAR CORR
  /VARIABLES=foldmiR155 folddmiR146 Bop suppuration PPD boneloss GW GTT
  /PRINT=SPEARMAN TWOTAIL NOSIG
  /MISSING=PAIRWISE.
```

```
 NONPAR CORR
  /VARIABLES=foldmiR155 folddmiR146 suppuration PPD boneloss GW GTT
  /PRINT=SPEARMAN TWOTAIL NOSIG
  /MISSING=PAIRWISE.
```

Nonparametric Correlations

Notes

Output Created		15-APR-2024 17:10:...
Comments		
Input	Data	/Volumes/Extreme SSD/new for review 2022-A/paper one/data paper one /paper one data.sav
	Active Dataset	DataSet1
	Filter	<none>
	Weight	<none>
	Split File	<none>
	N of Rows in Working Data File	16
Missing Value Handling	Definition of Missing	User-defined missing values are treated as missing.
	Cases Used	Statistics for each pair of variables are based on all the cases with valid data for that pair.
Syntax		NONPAR CORR /VARIABLES=foldmiR155 folddmiR146 suppuration PPD boneloss GW GTT /PRINT=SPEARMAN TWOTAIL NOSIG /MISSING=PAIRWISE.

Notes

Resources	Processor Time	00:00:00.01
	Elapsed Time	00:00:00.00
	Number of Cases Allowed	314572 cases [a]

a. Based on availability of workspace memory

Correlations

			foldmiR155	folddmiR146
Spearman's rho	foldmiR155	Correlation Coefficient	1.000	.544*
		Sig. (2-tailed)	.	.029
		N	16	16
	folddmiR146	Correlation Coefficient	.544*	1.000
		Sig. (2-tailed)	.029	.
		N	16	16
	suppuration	Correlation Coefficient	.058	-.292
		Sig. (2-tailed)	.831	.272
		N	16	16
	PPD	Correlation Coefficient	.076	-.007
		Sig. (2-tailed)	.778	.978
		N	16	16
	boneloss	Correlation Coefficient	-.059	-.136
		Sig. (2-tailed)	.828	.616
		N	16	16
	GW	Correlation Coefficient	.171	.252
		Sig. (2-tailed)	.526	.346
		N	16	16
	GTT	Correlation Coefficient	-.037	.085
		Sig. (2-tailed)	.892	.756
		N	16	16

Correlations

			suppuration	PPD	boneloss
Spearman's rho	foldmiR155	Correlation Coefficient	.058	.076	-.059
		Sig. (2-tailed)	.831	.778	.828
		N	16	16	16
	folddmiR146	Correlation Coefficient	-.292	-.007	-.136
		Sig. (2-tailed)	.272	.978	.616
		N	16	16	16
	suppuration	Correlation Coefficient	1.000	.062	.471
		Sig. (2-tailed)	.	.819	.065
		N	16	16	16
	PPD	Correlation Coefficient	.062	1.000	.709**
		Sig. (2-tailed)	.819	.	.002
		N	16	16	16
	boneloss	Correlation Coefficient	.471	.709**	1.000
		Sig. (2-tailed)	.065	.002	.
		N	16	16	16
	GW	Correlation Coefficient	-.259	-.141	-.170
		Sig. (2-tailed)	.333	.601	.530
		N	16	16	16
	GTT	Correlation Coefficient	-.065	.216	.211
		Sig. (2-tailed)	.812	.423	.433
		N	16	16	16

Correlations

			GW	GTT
Spearman's rho	foldmiR155	Correlation Coefficient	.171	-.037
		Sig. (2-tailed)	.526	.892
		N	16	16
	folddmiR146	Correlation Coefficient	.252	.085
		Sig. (2-tailed)	.346	.756
		N	16	16
	suppuration	Correlation Coefficient	-.259	-.065
		Sig. (2-tailed)	.333	.812
		N	16	16
	PPD	Correlation Coefficient	-.141	.216
		Sig. (2-tailed)	.601	.423
		N	16	16
	boneloss	Correlation Coefficient	-.170	.211
		Sig. (2-tailed)	.530	.433
		N	16	16
	GW	Correlation Coefficient	1.000	.323
		Sig. (2-tailed)	.	.222
		N	16	16
	GTT	Correlation Coefficient	.323	1.000
		Sig. (2-tailed)	.222	.
		N	16	16

*. Correlation is significant at the 0.05 level (2-tailed).

**. Correlation is significant at the 0.01 level (2-tailed).
